# Dysphagia and Oral Health in Older Adults with Motoric Cognitive Risk Syndrome

**DOI:** 10.1007/s00455-025-10849-9

**Published:** 2025-06-25

**Authors:** Özgü İnal Özün, Senem Demirdel, Necmiye Ün Yıldırım, Mehmet İlkin Naharci

**Affiliations:** 1 Department of Neurologic Physiotherapy and Rehabilitation, Gulhane Faculty of Physiotherapy and Rehabilitation, University of Health Sciences, Ankara, Turkey; 2 Department of Orthopedic Physiotherapy and Rehabilitation, Gulhane Faculty of Physiotherapy and Rehabilitation, University of Health Sciences, Ankara, Turkey; 3 Division of Geriatrics, Gulhane Faculty of Medicine & Gulhane Training and Research Hospital, University of Health Sciences, Ankara, Turkey

**Keywords:** Dysphagia, Motoric cognitive risk syndrome, Oral health, Subjective cognitive complaints

## Abstract

Slow gait speed and subjective cognitive decline in older adults are characteristics of motoric cognitive risk syndrome (MCRS). Dysphagia and oral health may be connected to MCRS because they are linked to both motor function and cognitive performance. This study aimed to investigate dysphagia and oral health among older adults with MCRS. Community-dwelling adults over 65 years of age who visited the geriatric outpatient clinic for regular check-ups were included (N = 152). Socio-demographic and clinical data were collected, and the Eating Assessment Tool (EAT-10) and Geriatric Oral Health Assessment Index (GOHAI) were implemented. Participants were divided into two groups as MCRS (N = 36) and non-MCRS (N = 116). Poorer GOHAI and EAT-10 scores were observed in the MCRS group (p < 0.05 for all). After adjusting for potential confounding factors, higher EAT-10 scores were found to be independently associated with MCRS (OR = 1.13, 95% CI: 1.04–1.23, p = 0.005), but not GOHAI scores. Our findings indicated an association between dysphagia and MCRS in older adults. This is the first study in the literature to examine the association between dysphagia and oral health among older adults with MCRS. MCRS is a very recent topic in the literature and the parameters associated with MCRS are not clear. This study will contribute to the literature filling an important gap because a better understanding of the mechanisms linking these two comorbidities is vital for the development of targeted interventions aimed at reducing swallowing difficulties in patients with MCRS.

## Introduction

Motoric cognitive risk syndrome (MCRS) is a newly proposed pre-dementia syndrome defined by subjective cognitive complaints (SCC) and slow walking speed without dementia [1]. Investigating the factors associated with MCRS is important for determining the parameters to focus on when developing preventive and therapeutic approaches [2].

Age-related changes in the mechanism of swallowing, defined as presbyphagia, occur with healthy aging [3]. Presbyphagia is caused by a variety of factors such as decreased salivary flow, dental issues, weakened pharyngeal and oral mucosal sensitivity, diminished brain compensating ability, and loss of muscle strength and function. These changes increase susceptibility to dysphagia, a condition in which it is difficult or uncomfortable to transfer food particles from the mouth to stomach and may act as precipitating factors [3, 4]. Dysphagia has a prevalence of 11–25% in community-dwelling older adults and up to 54% in those unable to live independently [5–7]. There is increasing evidence in the literature regarding the relationship between physical condition parameters and factors related to swallowing function in older adults. In different studies, risk factors associated with dysphagia were found to be related to physical frailty [6], tongue strength and grip strength, nutrition and oral intake status [7], tongue strength and grip strength [8], swallowing and whole body muscle strength and grip strength, and tongue pressure and grip strength and walking speed [9], dysphagia and chewing ability, dry mouth, physical function [10]. Furthermore, cognitive decline puts older adults at an increased risk of swallowing difficulties [11]. Therefore, the management of dysphagia in older age groups requires greater consideration of muscle function and cognitive assessments.

Oral health is negatively affected by aging due to factors such as periodontal disease and tooth loss [12]. In addition, a person’s eating habits and choices can be greatly impacted by poor oral health, which includes issues with swallowing, dry mouth (xerostomia) and excessive tooth loss [13, 14]. Poor diet such as low protein intake may adversely affect physical performance, muscle mass, and muscle strength at advanced ages [15, 16]. In a study conducted by Albani et al., while dry mouth symptoms, difficulty in swallowing, difficulty in eating, and tooth loss were associated with increased risks of mobility limitations, difficulty in eating was associated with weak grip strength and frailty. In addition, difficulty in eating was associated with increased risks of frailty, mobility limitations and slow gait speed. Finally, complete tooth loss was associated with increased risk of frailty [17]. In another study, a positive association between the number of teeth and mean gait speed was detected [18]. Thus, inadequate oral health increases the risk of physical and cognitive frailty in this population [19, 20].

One of the greatest threats to the health and independence of older people is MCRS, a condition prior to dementia [21, 22]. Studies have shown that both cognitive and physical impairments are associated with dysphagia and oral health problems [6–10]. Reduced cognitive functions may affect cognition or food intake during swallowing [23], which may be a factor that worsens dysphagia. A very recent study has shown that cognitive impairment has a causal effect on dysphagia from a genetic perspective and it has been pointed out that individuals with a history of cognitive impairment require special clinical attention to prevent the development of dysphagia [24]. Besides, there is plenty of evidence on the relationship between dysphagia and cognition. However, to the best of our knowledge, there is no study in the literature examining the relationship between dysphagia and subjective cognitive complaints (SCC). Since one of the diagnostic criteria of MCRS is SCC, the examination of dysphagia and oral health in MCRS may guide the relationship between SCC and these parameters. Dysphagia is also associated with motor functions. In addition, in research on independently living elderly people, a high prevalence of oropharyngeal dysphagia (OD) was found to be statistically associated with lower Barthel score, slower walking speed and lower overall quality of life (QoL). Thus, a close association was observed between OD symptoms and poor functional capacity [25]. As stated earlier, another diagnostic criterion for MCRS is slow walking speed. Considering the relationship between dysphagia and slow walking speed, it is thought that examining dysphagia in MCRS is important for the management of MCRS.

To our knowledge, there is no study reporting on the association of swallowing and oral health status with MCRS, which is a combination of cognitive complaints and slow gait. Thus, we hypothesized that dysphagia and poor oral health could be a predictor of MCRS. Therefore, this study aimed to investigate the relationship between these clinical conditions and MCRS in community-dwelling older adults.

## Materials and Methods

### Participants

This study included community-dwelling older adults who were admitted to the geriatric outpatient clinic for regular check-ups between July 2023 and July 2024. The inclusion criteria were as follows: (a) age ≥ 65 years, (b) ability to walk without an assistive device, (c) Mini-Mental State Examination (MMSE) score ≥ 24 points, (d) no neurological disease diagnosis (such as dementia, stroke, Parkinson disease, Amyotrophic Lateral Sclerosis), (e) no recent surgery, (f) independent oral feeding, and (e) literate. The exclusion criteria were as follows: (a) diagnosis of dementia, (b) wheelchair use, and (c) bedridden.

This study was conducted at the Health Sciences University Gülhane Training and Research Hospital. In compliance with the Declaration of Helsinki, the study received approval from the local ethics committee (Gülhane Scientific Research Ethics Committee (decision number: 2023-239)).

### Diagnosis of MCRS

The criteria proposed by Verghese et al. were used to diagnose MCRS [1]. According to these criteria, the participants were required to have the following three characteristics for the diagnosis of MCRS:

*(A) Subjective cognitive complaints (SCC):* individuals who answered “yes” to the Yesevage Geriatric Depression Scale question “Do you think you have more memory problems than most?” and those who performed normally on the MMSE (≥ 24 points) were considered to have probable subjective cognitive complaints [26].

*(B) Slow walking speed:* a stopwatch was used to measure the time taken to walk 4.57 m to determine the walking speed (m/s). Fried criteria, which necessitate stratification by height and gender, were used to classify participants as having a slow or normal walking speed [27].

*(C) Absence of dementia:* Those who did not have a diagnosis of dementia according to the Diagnostic and Statistical Manual of Mental Disorders Fifth Edition (DSM-5) criteria met this criterion.

**Fig. 1:**
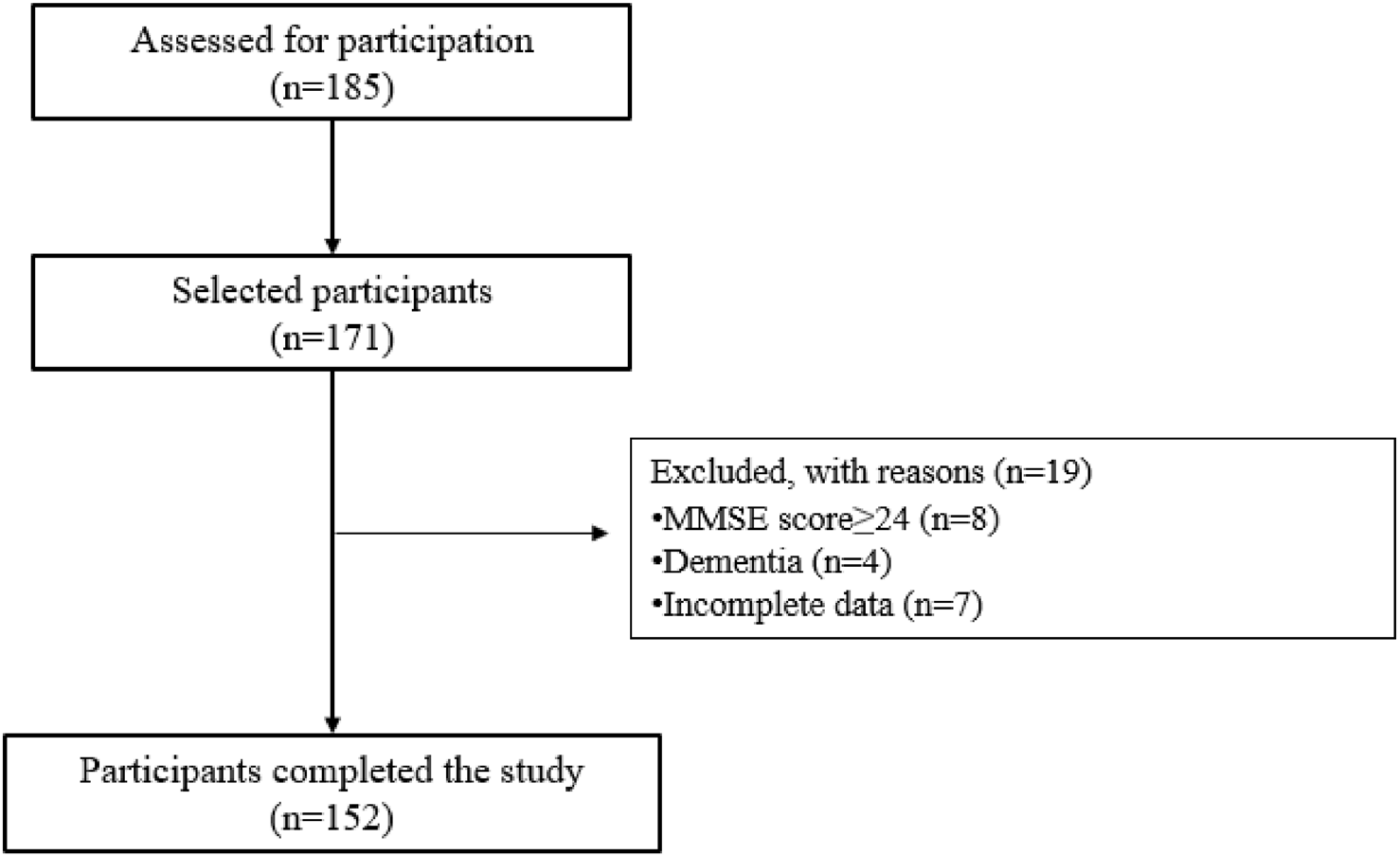
The flow of the sample selection process. MMSE: Mini Mental State Examination, MCRS: Motoric Cognitive Risk Syndrome

Following the exclusion of reversible causes of dementia and MCI, an experienced geriatrician arrived at the final clinical diagnosis, and the participants were split into two groups as those with and without a diagnosis of MCRS (MCRS and non-MCRS) (Fig. 1).

### Outcome Variables

Eating assessment tool (EAT-10) and Geriatric Oral Health Assessment Index (GOHAI) scores were outcome variables in examining the association between swallowing and oral health and MCRS.

*Eating Assessment Tool (EAT-10):* This scale is a self-administered questionnaire with 10 questions used to evaluate swallowing function [28]. Each question on the scale is answered on a 4-point scale (0 = no problem in swallowing function and 4 = severe problem in swallowing function). The total score of this scale ranges from 0 to 40, with higher scores indicating greater difficulty in swallowing [29].

*Geriatric Oral Health Assessment Index (GOHAI):* This scale, a self-report tool, with 12 questions to measure oral health status and define the physical functions of older adults (eating, swallowing, speaking), psychological functions and behavioral effects (concern or anxiety about oral health, external view, self-confidence, and social communication), and pain and discomfort (discomfort while eating, use of medication, etc.) on the last 3 months in older adults. The total score of GOHAI ranges from 0 to 60, with higher scores indicating better oral health [30, 31].

### Demographics and Other Variables

A sociodemographic form was used to record information about the participants obtained during the interviews and from their medical files. Age, gender, height, weight, level of education, presence of chronic diseases such as diabetes, hypertension and cardiac diseases, and history of falls in the last year were questioned. According to the participants’ statements, dental status (e.g., dental prosthesis and own tooth and/or implant) were recorded.

*Timed Up and Go Test (TUG):* This test is widely used to evaluate dynamic balance, gait speed, and mobility. The time it took for the person to get up from the chair, walk three meters, and then turn and sit back on the chair was recorded [32].

### Statistical Analysis

The IBM SPSS Statistics version 25.0 (IBM Corp., Armonk, NY, USA) was used for statistical analyses. Kolmogorov–Smirnov test and histogram were used to examine the conformity of the data to a normal distribution. Continuous variables were presented as median, Interquartile Range (IQR) and categorical variables as frequencies and percentages. In the comparison of individuals with and without MCRS, the Mann–Whitney U test and the chi-square (χ2) test were used for continuous variables and categorical variables, respectively. In the logistic regression analysis, MCRS was the dependent variable. Adjusted results for age, gender, education, the presence of chronic disease, EAT-10 and GOHAI scores were presented as odds ratios (OR) and 95% confidence intervals (CI). A significance level of p < 0.05 was deemed statistically significant. Post-hoc analysis power was performed in G Power 3.1.9.7 (MCRS group: 36, non-MCRS group: 116, power (1– β): 0.99).

## Results

The final sample included 152 subjects (mean age: 73.76 ± 6.80 years; 53.9% female). The prevalence of MCRS was 23.7% (N = 36). There was no statistically significant difference between the MCRS and non-MCRS groups in terms of demographic characteristics; the groups were similar (p < 0.05). Walking speed and GOHAI scores were lower, whereas EAT-10 scores and TUG times were higher in the MCRS group than in the non-MCRS group (all p < 0.05) (Table 1).

**Table 1:** Demographic and clinical data of the participants

Demographics	Total (N = 152)	MCRS (N = 36)	Non-MCRS (N = 116)	p
Age (years)				
65–75 years	90 (59.2)	17 (47.2)	73 (62.9)	0.094
≥ 75 years	62 (40.8)	19 (52.8)	43 (37.1)	
Gender (female)	82 (53.9)	20 (55.5)	62 (53.4)	0.825
Education (≤ 5 years)	78 (51.3)	19 (52.7)	59 (50.8)	0.841
Presence of chronic disease (yes)	125 (82.2)	31 (86.1)	94 (81.0)	0.486
Falls within the last 1 year (yes)	59 (38.8)	13 (36.1)	46 (39.6)	0.703
BMI (kg/m^2^)				
18.5–24.99	50 (32.9)	10 (27.8)	40 (34.5)	0.456
≥ 25	102 (67.1)	26 (72.2)	76 (65.5)	
Dental condition				
Dental prothesis (partial or total)	101 (66.4)	23 (63.9)	78 (67.2)	0.931
Own tooth and/or implant	51 (33.6)	13 (36.1)	38 (32.8)	
Outcome variables				
Walking speed (m/s), median (IQR)	0.73 (0.6–0.91)	0.53 (0.42–0.62)	0.83 (0.69–0.98)	≤ 0.001*
MMSE (0–30), median (IQR)	26.9 (24–30)	26.6 (24–30)	27.0 (24–30)	0.214
EAT-10 (0–60), median (IQR)	3.1 (0.0–21.0)	5.8 (9.5–0.8)	0.8 (2.9–0.0)	≤ 0.001*
GOHAI (12–60), median (IQR)	52.1 (20.0–60.0)	50.6 (58.2–40.0)	56.7 (59.4–49.9)	0.005*
TUG (sec), median (IQR)	11.8 (5.5–37.7)	14.3 (17.6–11.5)	9.6 (12.0–8.0)	≤ 0.001*

MCRS: motoric cognitive risk syndrome, MMSE: Mini Mental State Examination, TUG: Timed Up and Go Test, *EAT-10:* Eating Assessment Tool, GOHAI: Geriatric Oral Health Assessment Index, IQR: Interquartile Range 1–3

*Values are statistically significant results (p < 0.05)

Table 2 indicates the potential association of variables to the risk of MCRS. In adjusted logistic regression analysis, each point increase in EAT-10 scores was associated with MCRS (OR = 1.13; 95% CI:1.04–1.23; p = 0.005). According to multivariable regression analysis, age, gender, education, presence of chronic disease, oral health status did not achieve statistical significance.

**Table 2:** Logistic regression model to estimate the effect of age, gender, education, presence of chronic disease, swallowing function and oral health on MCRS

Variables	B	O.R.	OR(95%CI)		p
			Lower	Upper	
Age	0.412	1.509	0.665	3.423	0.325
Gender	− 0.061	0.941	0.392	2.257	0.891
Education	− 0.147	0.863	0.357	2.088	0.744
Chronic disease	− 0.358	0.699	0.226	2.164	0.535
EAT-10	0.123	1.134	1.038	1.233	0**.005**
GOHAI	− 0.034	0.966	0.925	1.009	0.120

Bold value indicates statistically significant results (p < 0.05)

EAT-10: Eating Assessment Tool, GOHAI: Geriatric Oral Health Assessment Index, CI: Confidence Intervals

The Hosmer–Lemeshow test, an inferential goodness of fit test, produced a Chi-Square of 4.739 and was found to be non-significant (p > 0.05), indicating that the model fits the data well.

## Discussion

To the best of our knowledge, this is the first study in the literature to examine the association between dysphagia and oral health among older adults with MCRS. We observed that swallowing difficulties were independently linked to MCRS. This outcome is original and merits consideration in many aspects. In addition, one in every four older individuals living in the community had MCRS. However, it was found that the oral health status was not related to the risk of MCRS. Therefore, our findings may contribute to efforts to improve the quality of healthcare by implementing preventive approaches in older adults with MCRS who are at risk for falls, disability, dementia, and mortality.

The prevalence of MCRS varies depending on ethnicity, educational level and economic sources. In community-based cross-sectional studies, the prevalence of MCRS ranges from 1.76 to 27% [33–35]. MCRS prevalence rates are lower in high-income countries, but how MCRS criteria are operationalized across studies also influences rates [35]. We found a relatively high prevalence of MCRS. Given that individuals with cognitive or physical disabilities presenting to a referral center due to medical problems were more likely to participate in our study, the increased prevalence of MCRS may be the result of a selection bias [34].

Dysphagia is a complex condition involving the oral, oropharyngeal, and esophageal swallowing phases due to a wide variety of etiologies and co-occurring conditions. Dysphagia is a geriatric syndrome, but other age-related clinical conditions may also produce problems with safe eating and swallowing [36–38]. Frailty, a clinical condition of vulnerability, or any of its constituents (e.g., low handgrip strength and gait speed) may place older adults at an increased risk of dysphagia [38, 39]. This association implies that in addition to muscle strength, dysphagia is associated with other aspects of mobility, such as walking speed and balance [40]. In support in the central nervous system (CNS) [e.g., cerebellum, cortical motor and premotor areas, and central pattern generator (CPG)] control them [41, 42]. For example, the CPG generates motor commands during mastication based on sensory information from muscle spindles on either side of the jaw-closing muscles and periodontal mechanoreceptors. Sensory information from mechanoreceptors and lower limb muscle spindles regulates walking-related CPG [41]. Furthermore, it has been suggested that the locomotor-respiratory coupling (LRC) in quadrupeds—the synchronization of breathing and walking/running rhythms—is supported by a visceral piston mechanism. According to this idea, the lungs are rhythmically compressed by the visceral mass when moving [43]. In a study investigating potential mechanisms underlying the chewing-walking link [41], it was determined that the involvement of the habitual chewing rhythm in walking speed is a potentially important phenomenon in humans, as the dominant ratio of chewing-walking-head movement rhythms is 2:1:2 in most of the healthy volunteers. The literature has also demonstrated that during walking, changes in the mandible’s position in relation to the maxilla can result in stretch reflexes in the jaw muscles [44, 45]. In our study, higher dysphagia scores were obtained in the MCRS group. Considering slow gait, which is one of the diagnostic criteria of MCRS, this may be related to both the mechanisms between head movements and gait, and similar neural control points of impairment in walking and swallowing functions. However, more research is needed to support this hypothesis and explain this mechanism. Identifying the problems that can be seen in individuals with MCRS is critical for a better understanding of MCRS and better management of the disease process.

There is also the possibility of a bidirectional interaction between dysphagia and MCRS. MCRS is a predementia syndrome and dysphagia is common in dementia. More than half of individuals diagnosed with dementia may have difficulties in eating, drinking and swallowing [46]. A recent meta-analysis of 12,532 older adults with different dementia subtypes found a pooled prevalence of swallowing disorders among older adults with dementia of 58% [47]. Additionally, dysphagia is a significant predictor of poorer clinical and operational outcomes such as a 38% longer length of stay in patients hospitalized for dementia [48]. It has been shown that cognitive deficits found in neurological diseases such as Alzheimer’s dementia can cause disruption of the presenting and preparatory actions required for swallowing [49]. Subjective cognitive complaints (SCC) is present in individuals with MCRS. The literature suggests that some individuals with SCC have preclinical evidence of Alzheimer’s disease and therefore have a higher risk of progression to dementia [50]. Although the current literature provides evidence for the relationship between SCC, one of the diagnostic criteria of MCRS, and dysphagia, further research should be conducted on this subject to explain this bidirectional interaction.

Poor oral health is common among older adults. Current findings emphasize that oral health status may contribute significantly to general well-being, including Alzheimer’s disease and cognitive impairment in late life [51]. Moreover, there is evidence that people with dementia have more gingival and periodontal difficulties, which are related to the severity of their dementia [52]. Poor oral hygiene has also been associated with a decline in cognitive function in individuals with dementia [53]. A pre-dementia disease known as MCRS combines cognitive symptoms (self-reported cognitive complaints) with motor indicators (slow gait) [54]. Therefore, it can be asserted that having MCRS increases the risk of dementia and other adverse age-related outcomes [35]. In this study, although there was a difference in oral health between the MCRS and non-MCRS groups, this disappeared in multivariate regression analysis. In this study, GOHAI was used to assess the oral health of individuals. In future studies, non-significant GOHAI results should be further investigated in terms of possible confounding factors or limitations of the questionnaire. In addition, the study’s limited sample size and questionnaire-based evaluation of oral health may explain the lack of a relationship between oral health and MCRS. Oral health was assessed with a questionnaire in this study and parameters such as periodontal problems and number of teeth were not evaluated. Thus, it can be concluded that more detailed future studies evaluating oral health will provide a better understanding of oral health in individuals with MCRS.

The reason for the variation in the risk factors for MCRS is that research on this topic is limited, and variables related to sample size and age vary among studies. Although most studies used 60, 65, or 70 years as the entry age, only two studies included people aged 75 [55, 56]. In another recently published study, it was reported that polypharmacy and older age were risk factors associated with MCRS, while gender, education status, and comorbidity burden were not risk factors for MCRS [33]. A recent meta-analysis identified novel associations between MCRS and poor sleep, hearing, poor cognition, and multiple falls. When stratified by income, some risk factors (e.g., education) were associated with the MCRS in high- and middle-income countries. Others (e.g., obesity) were specific to high-income countries [57]. The same study noted that, contrary to the literature, some cardiovascular risk factors (such as diabetes and hypertension) that are consistently linked with MCRS were not associated with MCRS in all countries. Considering the available evidence in the literature, it can be concluded there is a need for clinical evaluations with a broader perspective to determine the risk factors associated with MCRS.

In diseases such as dementia, interventions will be most effective as early as possible in the disease process, when there is minimal pathology and fewer behavioral and cognitive symptoms. This applies to both pharmacological treatments and non-pharmacological therapies such as cognitive-behavioral memory groups and risk reduction interventions [58, 59]. Given that there are many unknown aspects of MCRS, early diagnosis and interventions in MCRS require knowledge of the risks associated with MCRS and possible associated risk factors. In this context, it is thought that the results obtained from the current study will guide the early determination of patients with MCRS, the knowledge of the factors associated with MCRS, and the management of MCRS.

We acknowledge that our study has some limitations. First, the cross-sectional design of this study did not reveal any causality. However, we aim to analyze the data obtained through longitudinal follow-up in the future and share interpretations of the results. Second, we did not apply an objective method such as videofluoroscopy and/or FESS to detect dysphagia [60, 61]. The study is based on self-reported instruments (EAT-10 and GOHAI) which have limitations such as subjective bias. It is important to use instruments with objective assessments in future studies to strengthen the results. To overcome this limitation, a physiotherapist specializing in dysphagia implemented the EAT-10, which has excellent consistency and validity [29, 62]. Third, although we had adequate power with the number of participants in the groups, our sample size was smaller than those of the studies examining the factors associated with MCRS. Lastly, we acknowledge that there may be unmeasured or residual confounding factors in MCRS risk.

## Conclusion

Although MCRS and swallowing difficulties are common in older adults, this study also demonstrated a link between these two comorbidities. Therefore, it is thought that clinicians may benefit from the results of our study to identify the factors associated with MCRS, allowing early detection of these patients and appropriate therapeutic approaches to prevent progression of the disease. Finally, we suggest that a clinical approach that objectively examines swallowing function in routine assessment models is needed for further research on the risk of dysphagia in older adults with MCRS.

## Data Availability

The data set generated and analyzed during the current study can be obtained from the corresponding author upon reasonable request.
