# Correction: Dysphagia and Oral Health in Older Adults with Motoric Cognitive Risk Syndrome

**DOI:** 10.1007/s00455-025-10886-4

**Published:** 2025-09-18

**Authors:** Özgü İnal Özün, Senem Demirdel, Necmiye Ün Yıldırım, Mehmet İlkin Naharci

**Affiliations:** 1 Department of Neurologic Physiotherapy and Rehabilitation, Gulhane Faculty of Physiotherapy and Rehabilitation, University of Health Sciences, Ankara, Turkey; 2 Department of Orthopedic Physiotherapy and Rehabilitation, Gulhane Faculty of Physiotherapy and Rehabilitation, University of Health Sciences, Ankara, Turkey; 3 Division of Geriatrics, Gulhane Faculty of Medicine & Gulhane Training and Research Hospital, University of Health Sciences, Ankara, Turkey

**Correction to: Dysphagia**

**https://doi.org/10.1007/s00455-025-10849-9**

In Table 1 of this article, some numerical values were inadvertently placed in incorrect cells 1 and has now been corrected in the original publication. For completeness and transparency, the old incorrect versions and correct version are displayed below.

**Incorrect version**

**Table 1:** Demographic and clinical data of the participants

Demographics	Total (N = 152)	MCRS (N = 36)	Non-MCRS (N = 116)	p
Age (years)				
65–75 years	90 (59.2)	17 (47.2)	73 (62.9)	0.094
≥ 75 years	62 (40.8)	19 (52.8)	43 (37.1)	
Gender (female)	82 (53.9)	20 (55.5)	62 (53.4)	0.825
Education (≤ 5 years)	78 (51.3)	19 (52.7)	59 (50.8)	0.841
Presence of chronic disease (yes)	125 (82.2)	31 (86.1)	94 (81.0)	0.486
Falls within the last 1 year (yes)	59 (38.8)	13 (36.1)	46 (39.6)	0.703
BMI (kg/m^2^)				
18.5–24.99	50 (32.9)	10 (27.8)	40 (34.5)	0.456
≥ 25	102 (67.1)	26 (72.2)	76 (65.5)	
Dental condition				
Dental prothesis (partial or total)	101 (66.4)	23 (63.9)	78 (67.2)	0.931
Own tooth and/or implant	51 (33.6)	13 (36.1)	38 (32.8)	
Outcome variables				
Walking speed (m/s), median (IQR)	0.73 (0.6–0.91)	0.53 (0.42–0.62)	0.83 (0.69–0.98)	≤ 0.001*
MMSE (0–30), median (IQR)	26.9 (24–30)	26.6 (24–30)	27.0 (24–30)	0.214
EAT-10 (0–60), median (IQR)	3.1 (0.0–21.0)	5.8 (9.5–0.8)	0.8 (2.9–0.0)	≤ 0.001*
GOHAI (12–60), median (IQR)	52.1 (20.0–60.0)	50.6 (58.2–40.0)	56.7 (59.4–49.9)	0.005*
TUG (sec), median (IQR)	11.8 (5.5–37.7)	14.3 (17.6–11.5)	9.6 (12.0–8.0)	≤ 0.001*

MCRS: motoric cognitive risk syndrome, MMSE: Mini Mental State Examination, TUG: Timed Up and Go Test, *EAT-10:* Eating Assessment Tool, GOHAI: Geriatric Oral Health Assessment Index, IQR: Interquartile Range 1–3

*Values are statistically significant results (p < 0.05)

**Correct version**

**Table 1:** Demographic and clinical data of the participants

**Demographics**	**Total** **(N=152)**	**MCRS** **(N=36)**	**Non-MCRS** **(N=116)**	**p**
Age (years)				
65-75 years	90 (59.2)	17 (18.9)	73 (81.1)	0.094
≥75 years	62 (40.8)	19 (30.6)	43 (69.4)	
Gender (female)	82 (53.9)	20 (24.4)	62 (75.6)	0.825
Education (≤ 5 years)	78 (51.3)	19 (24.4)	59 (75.6)	0.841
Presence of chronic disease (yes)	125 (82.2)	31 (24.8)	94 (75.2)	0.486
Falls within the last 1 year (yes)	59 (38.8)	13 (22.04)	46 (77.96)	0.703
BMI (kg/m²)				
18.5-24.99	50 (32.9)	10 (20.0)	40 (80.0)	0.456
≥25	102 (67.1)	26 (25.5)	76 (74.5)	
Dental condition				
Dental prothesis (partial or total)	101 (66.4)	23 (22.7)	78 (77.3)	0.931
Own tooth and/or implant	51 (33.6)	13 (25.5)	38 (74.5)	
Outcome variables				
Walking speed (m/s), median (IQR)	0.73 (0.6-0.91)	0.53 (0.42-0.62)	0.83 (0.69-0.98)	≤0.001*
MMSE (0-30), median (IQR)	26.9 (24-30)	26.6 (24-30)	27.0 (24-30)	0.214
EAT-10 (0-60), median (IQR)	3.1 (0.0-21.0)	5.8 (9.5-0.8)	0.8 (2.9-0.0)	≤0.001*
GOHAI (12-60), median (IQR)	52.1 (20.0-60.0)	50.6 (58.2-40.0)	56.7 (59.4-49.9)	0.005*
TUG (sec), median (IQR)	11.8 (5.5-37.7)	14.3 (17.6-11.5)	9.6 (12.0-8.0)	≤0.001*

MCRS: motoric cognitive risk syndrome, MMSE: Mini Mental State Examination, TUG: Timed Up and Go Test, *EAT-10*: Eating Assessment Tool, GOHAI: Geriatric Oral Health Assessment Index, IQR: Inter Quantile Range 1–3

*Values are statistically significant results (*p* < 0.05)

